# FOURTH INTERNATIONAL CONGRESS ON OXYGEN RADICALS (4–ICOR)

**DOI:** 10.6028/jres.092.034

**Published:** 1987-10-01

**Authors:** Michael Baum

**Affiliations:** Public Information Division, National Bureau of Standards, Gaithersburg, MD 20899

More than 400 scientists and researchers from around the world at the Fourth International Congress on Oxygen Radicals in La Jolla, CA, last June considered the less benign effects of breathing oxygen.

Among the 200-plus papers presented at the 4-ICOR meeting organized by the NBS were those that: strengthened current theories on the importance of vitamins E and C in the diet, advocated the increase of fish and decrease of vegetable oil in the American diet, provided direct evidence of oxygen radical involvement in damage to organs that are resupplied with blood after being cut off from blood supply, and questioned the value of at least one high-tech health fad, hyperbaric (high- pressure) oxygen therapy.

Subtle biochemical mechanisms that may one day lead to prevention and new therapies to. combat atherosclerosis, cancer, arthritis, various eye disorders, and an expanding list of other diseases, as well as the general effects of aging, were also discussed.

The last several years have seen an increasing awareness among chemists, biochemists, research physicians, and others of the key role played in biochemistry by “oxygen radicals,” a variety of highly active chemical species derived from oxygen.

Although the idea of oxygen radicals has been around in one form or another since the 19th century, they have only been seriously considered in biochemistry since the 1960s, after researchers Joseph McCord and Irwin Fridovich of Duke University demonstrated the existence of a naturally occurring enzyme, superoxide dismutase (SOD) which seems to have no purpose other than to convert the simple oxygen radical to the more stable forms of hydrogen peroxide and molecular oxygen. The existence of SODs suggests that the superoxide radical, at least, is so prevalent in biochemical systems that enzymes have evolved to deal with it.

A related species, “singlet oxygen,” was discovered in the early 1960s. Though not technically a radical, singlet oxygen spurred the study of the oxidation of organic and biomolecular systems. Radiation researchers contributed information on the extremely reactive hydroxyl radical to the growing list of oxygen species.

Since then, mushrooming research activity around the world has built up a picture of oxygen radicals intimately involved in a host of biochemical processes, both good and bad.

In brief, short-lived radicals are now believed to be a generally toxic by-product of cell processes, but with occasional benefits. One paper presented at this meeting by Kelvin J. A. Davies and Susan McKenna from the University of Southern California, for example, offers a new explanation of the mechanism by which “neutrophils” in the blood attack and destroy invading bacteria. According to this theory, the neutrophils initiate a rapid chain of reactions, beginning with the conversion of molecular oxygen to superoxide radicals and ending with the production of hypochlorous acid–common laundry bleach. This “bleach” then attacks the DNA and RNA synthesis mechanisms in the bacteria, destroying their ability to reproduce. This allows the neutrophils to dismantle it at leisure.

In general, the body seems to have a variety of natural mechanisms to keep oxygen radicals under control, transmuting them to other, less reactive forms and making use of them along the way. The system exists in a complex state of balance. Unfortunately, these mechanisms sometimes go awry, and it was the aim of the researchers gathered at 4-ICOR to understand how and why.

A growing study of evidence, much of it discussed at this meeting, implicates uncontrolled free radicals in a variety of diseases, including the two major killers in Western nations: atherosclerosis and cancer. Although still somewhat controversial, an increasing number of researchers believe that many of the basic effects of aging are due to the gradual accumulation of the products of reactions of oxygen radicals with cell components.

Some items raised at 4-ICOR include:

– Vitamins E and C are increasingly seen to be important in protecting cells from the harmful effects of oxygen radicals. Vitamin E, embedded in the cell’s membranes, sacrifices itself to break an otherwise cell-lethal chain reaction initiated by oxygen radicals. Vitamin C, found in fluids outside the cell, sacrifices itself to repair the damaged vitamin E.
– The importance of regulating the *kinds* of fats in the diet is receiving increasing attention, according to University of Illinois researcher William Lands. By far the most common fats in the American diet are so-called “n-6 fatty acids,” found in almost all vegetable oils, including corn, soybean, and safflower, and in the flesh of animals fed those grains. Although important to the body’s mechanism of cell messenger molecules, says Lands, an excessive imbalance of these oils, and their biological products, has been implicated in a rogues’ gallery of diseases ranging from headaches, menstrual cramps and hypertension through heart disease, stroke, coronary thrombosis, and cancer. Many of these effects apparently can be mitigated, according to Lands, by diminishing the use of n-6-type oils and increasing the intake of n-3-type oils found in green, leafy vegetables and, in particular, seafoods. “What’s particularly impressive, I think,” says Lands, “is that every single institute in the National Institutes of Health has requested researchers to explore this theory.”
– Researchers working with Gregory Bulkley from Johns Hopkins University presented what may be the first direct evidence in a living system of the action of oxygen radicals in damaging organs that have been deprived of blood and then resupplied. This phenomenon, called “ischemia (blood deprivation) and reperfusion (resupply)” has received much attention recently because it seems to be one of the primary ways in which the brain, heart, and other organs are damaged after strokes, heart attacks, surgery, or other events that temporarily halt blood supply. Oxygen free radicals have been the chief suspects in the process, and the Johns Hopkins team reports the detection of the extremely low levels of light that are generated by free radical reactions, called chemiluminescence, in intact, living organs at reperfusion. Drugs that limit the action of the radicals in these cases may dramatically improve the chances of stroke victims to achieve full recovery.
– Hyperbaric oxygen chambers which involve breathing almost pure oxygen under pressure is seen by some researchers here as needlessly risky except in the treatment of certain conditions such as gas gangrene or stroke. Elevated levels of oxygen at high pressures bring on convulsions and death in lab animals, according to University of New South Wales researcher Dana Jamieson, with oxygen radical-mediated processes in the lung and brain vying to bring on death. “If you think of oxygen as a drug,” observes Jamieson, “it has a very low therapeutic ratio. It would be difficult or impossible to get it by the FDA.”
– Exactly how much exercise is good for you may be a more complicated question than generally realized, according to University of California researcher Lester Packer, who presented data to the congress showing that exercisers in training can develop temporarily decreased levels of vitamin E in their cells. Packer’s group also presented the first preliminary evidence of a free-radical reductase, an enzyme which can repair vitamin E after it has been damaged by an oxygen radical.

The first International Congress on Oxygen Radicals was organized 10 years ago in Canada in an effort to bring together researchers from a wide variety of fields, including physical chemistry, physics, biomedicine, and medical research, to pool information on the behavior of oxygen radicals and their products. Subsequent congresses were held in Texas and West Germany.

One decision made at the current meeting was to form a new society–tentatively called “The Oxygen Society”–to encourage interdisciplinary research and discussion on the general topic of oxygen biochemistry. The society would complement the existing Society for Free Radical Research founded in Europe.

The Fourth International Congress on Oxygen Radicals, held June 28–July 3,1987, was also sponsored by the National Heart, Lung, and Blood Institute; the International Life Sciences Institute; the National Cancer Institute; the American Industrial Health Council; and the Institute for Research on Aging. Seventeen major corporations and industrial associations also contributed to the congress.

